# Age-Dependent Decline in Synaptic Mitochondrial Function Is Exacerbated in Vulnerable Brain Regions of Female 3xTg-AD Mice

**DOI:** 10.3390/ijms21228727

**Published:** 2020-11-19

**Authors:** César Espino de la Fuente-Muñoz, Mónica Rosas-Lemus, Perla Moreno-Castilla, Federico Bermúdez-Rattoni, Salvador Uribe-Carvajal, Clorinda Arias

**Affiliations:** 1 Departamento de Medicina Genómica y Toxicología Ambiental, Instituto de Investigaciones Biomédicas, Universidad Nacional Autónoma de México, Ciudad de México 04510, Mexico; cesarespinofm@hotmail.com; 2 Departamento de Genética Molecular, Instituto de Fisiología Celular, Universidad Nacional Autónoma de México, Ciudad de México 04510, Mexico; monicarosaslemus@gmail.com (M.R.-L.); suribe@ifc.unam.mx (S.U.-C.); 3 Departmento de Neurociencia Cognitiva, Instituto de Fisiología Celular, Universidad Nacional Autónoma de México, Ciudad de México 04510, Mexico; perla.moreno.castilla@gmail.com (P.M.-C.); bermudez@unam.mx (F.B.-R.)

**Keywords:** synaptic mitochondria, brain aging, synaptosomes, mitochondrial dynamics, amyloid-β protein, tau

## Abstract

Synaptic aging has been associated with neuronal circuit dysfunction and cognitive decline. Reduced mitochondrial function may be an early event that compromises synaptic integrity and neurotransmission in vulnerable brain regions during physiological and pathological aging. Thus, we aimed to measure mitochondrial function in synapses from three brain regions at two different ages in the 3xTg-AD mouse model and in wild mice. We found that aging is the main factor associated with the decline in synaptic mitochondrial function, particularly in synapses isolated from the cerebellum. Accumulation of toxic compounds, such as tau and Aβ, that occurred in the 3xTg-AD mouse model seemed to participate in the worsening of this decline in the hippocampus. The changes in synaptic bioenergetics were also associated with increased activation of the mitochondrial fission protein Drp1. These results suggest the presence of altered mechanisms of synaptic mitochondrial dynamics and their quality control during aging and in the 3xTg-AD mouse model; they also point to bioenergetic restoration as a useful therapeutic strategy to preserve synaptic function during aging and at the early stages of Alzheimer’s disease (AD).

## 1. Introduction

Aging is associated with a variety of brain impairments that range from a slight decline in cognitive performance to severe memory loss and is the main risk factor for neurodegenerative diseases. In healthy aging, slowed neurotransmission and reduced synaptic plasticity have been reported [1,2,3]. However, not all brain regions are affected to the same extent during aging. For example, the cerebellum, hippocampus, and frontal cortex present a more evident volume reduction than other areas, possibly due to several factors, such as reduced dendritic branching, neuronal shrinkage, and synaptic loss [4,5]. In Alzheimer´s disease (AD), synaptic terminal loss precedes neuronal death and is the best correlate with cognitive decline [6,7,8,9,10]. The mechanisms governing the particular susceptibility of synaptic endings to aging and neurodegeneration are only now becoming clear and indicate the participation of synaptic mitochondria as a central player [11]. Emerging evidence suggests that reduced synaptic bioenergetics may be an early event that compromises neurotransmission and synaptic integrity in vulnerable brain regions [1,2,12,13].

Energy supply from mitochondria is essential to sustain metabolic demands and signaling functions at the synapsis, making mechanisms that regulate mitochondrial biogenesis and dynamics a crucial area of study to understand how these processes participate in synaptic dysfunction in AD. Synaptic mitochondria arrive from the neuronal soma via anterograde transport mediated by a variety of proteins involved in mitochondrial maintenance and dynamics [14]. Through mitochondrial fusion, an exchange of mitochondrial contents occurs, which is essential to preserve mitochondrial energy capacity and for the distribution of these organelles in neurons [15]. Interestingly, mutation in the mitochondrial fusion gene Mfn2 causes the neurodegenerative disease Charcot-Marie-Tooth disease [16]. Mitochondrial fission through dynamin-related protein 1 (Drp1) can help mitochondrial degradation in Parkin1-mediated mitophagy [17]. Recent work found a protective effect against amyloid toxicity on synapses and mitochondria by reducing the expression of Drp1 [18,19]. This evidence strongly suggests the critical role of mitochondrial dynamics and their regulation by fission and fusion proteins to preserve mitochondrial activity in neurons and synapses.

A reduced number of mitochondria have been reported in the human brain with AD [20], as well as an imbalance in the contents of fusion/fission proteins [18,21,22,23]. Similarly, an increase in the fission protein Fis1 is associated with a high rate of mitochondrial fragmentation in neuroblastoma cells that overexpress the mutated human amyloid precursor protein (APP) [24]. In the transgenic mouse model of AD overexpressing APP/Aβ, fragmented mitochondria with decreased activity of cytochrome oxidase C were also reported [25]. Furthermore, studies in isolated mitochondria from the triple transgenic mouse model (3xTg-AD) indicate bioenergetic dysfunction [26] similar to those found in the transgenic rat model that overexpressed mutated human APP [27]. There is a clearly emerging link between neurodegeneration and defects in mitochondrial dynamics and bioenergetics. Most of these studies were performed with neurons or mitochondria isolated from brain extract except for some recent work in synaptosomes isolated from brain cortices that reported mitochondrial dysfunction in aged 5xFAD mice [28]. 

Thus, to shed light on whether there is a relationship between aging and AD in mitochondrial synaptic dysfunction, we conducted experiments in isolated presynaptic terminals from three brain regions implicated in AD pathology from female 3xTg-AD mice and female wild-type (WT) mice at young and middle age, at which there are no substantial changes in presynaptic number but in synaptic functionality [7,8]. We analyzed the levels of proteins related to mitochondrial dynamics, mitochondrial bioenergetics, and mitochondrial ultrastructure. We found an important effect of aging; animals at 10 months but not 3 months old showed decreased mitochondrial bioenergetics that was slightly exacerbated in animals carrying the mutated proteins expressed in the 3xTg-AD 10-month-old mice. The observed changes consisted of a significant reduction in mitochondrial bioenergetics and mitochondrial membrane potential accompanied by an increase in the content of the activated form of Drp1 and intramitochondrial accumulation of the Aβ peptide and tau protein.

## 2. Results

### 2.1. Evaluation of Mitochondrial Redox Capacity

**Figure 1:**
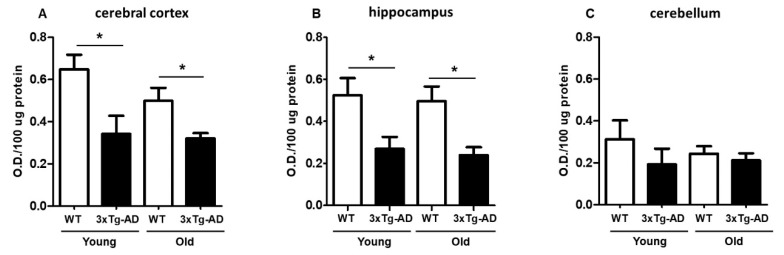
Mitochondrial redox capacity of synaptosomes. Synaptosomes were isolated from young and middle-aged female wild-type (WT) and 3xTg-AD mice and were assessed by MTT reduction after a 90 min incubation. Cortical synaptosomes (**A**), hippocampal synaptosomes (**B**) and cerebellar synaptosomes (**C**) are shown. Data are expressed as the mean percentage ± SEM of duplicates from 3–4 different individual animals per group. Student’s t test * *p* < 0.05.

The extent of MTT reduction was considered an index of synaptic viability and mitochondrial redox activity, as we previously described [29,30]. We first examined the age-dependent change in mitochondrial redox activity in synaptosomes isolated from the cerebral cortex, hippocampus, and cerebellum from young and aged WT and 3xTg-AD mice (Figure 1). As shown in Figure 1A, a significant decrease in MTT reduction in cortical synaptosomes was observed in young 3xTg-AD mice compared with young WT mice (40% inhibition) and in aged WT mice compared with aged 3xTg-AD mice (30% inhibition). A similar effect was also found in hippocampal synaptosomes from young and aged mice (53% inhibition) (Figure 1B). Finally, the synaptosomes isolated from the cerebellum, a region that was not expected to accumulate AD-related markers, did not present significant differences between WT and 3xTg-AD at any age, but the basal MTT reduction capacity seemed to be lower in this region than in the hippocampus or cerebral cortex (Figure 1C).

### 2.2. Reduced Mitochondrial Bioenergetics Associated with Age

**Figure 2:**
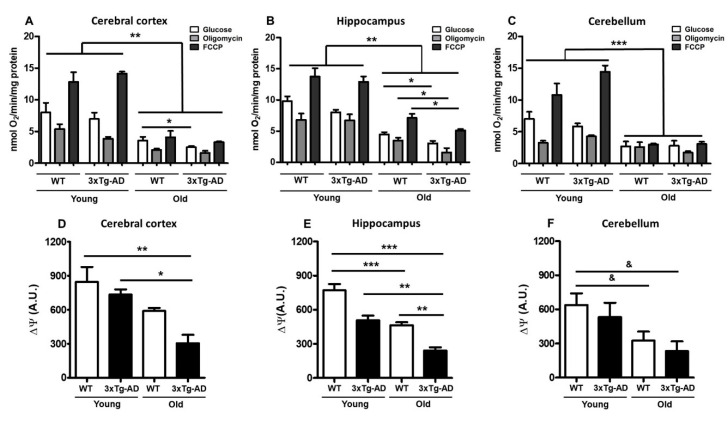
Age-related changes in oxygen consumption and mitochondrial membrane potential. Mitochondrial bioenergetics was measured in synaptosomes isolated from young and old female 3xTg-AD and WT mice. The oxygen consumption rate of cortical (**A**), hippocampal (**B**), and cerebellar (**C**) synaptosomes was measured in the presence of 10 mM glucose (white bar), 6 μM oligomycin (gray bar), or 1 μM FCCP (black bar). Values are expressed as nmol O_2_/min/mg protein and are the mean percentage ± SEM of duplicate measurements from 3 independent animals per group. Fluorescence changes in the mitochondrial membrane potential probe Safranin O in cortical (**D**), hippocampal (**E**), and cerebellar (**F**) synaptosomes are shown. Arbitrary units are the mean ± SEM from triplicate measurements of 3–4 animals per group. ANOVA and Tukey’s multiple comparison test, * *p* < 0.05; ** *p* < 0.01; *** *p* < 0.001. Student’s t test, ^&^ *p* < 0.05.

To evaluate the mitochondrial respiration rate at different ages, we measured basal oxygen consumption and respiratory coupled control in nonpermeabilized synaptosomes; then, we evaluated the effect of glucose addition and after exposure to the ATP synthase inhibitor oligomycin and the oxidative phosphorylation uncoupler FCCP. In the cerebral cortex, healthy synaptic mitochondria from young WT mice exhibited 8.0 nanomoles of basal O_2_ consumption and 5.3 or 12.8 after oligomycin or FCCP treatment, respectively—very similar to that observed in young 3xTg-AD mice (6.9, 3.9 and 14.1, respectively). However, compared to WT young synaptosomes, synaptosomes from old WT mice showed significantly reduced oxygen consumption rates under the three tested conditions (3.5, 2.1 and 4, respectively). An additional and significant reduction was observed in synaptosomes from old 3xTg-AD mice compared with those from old WT mice (2.5, 1.62, and 3.3, respectively) (Figure 2A, Table 1). Synaptic mitochondria from the hippocampus presented a similar pattern of oxygen consumption to that observed in synaptic mitochondria from the cerebral cortex. In young animals, oxygen consumption was similar between WT and 3xTg-AD mice (9.8, 6.7, and 13 nmol O_2_ in WT vs. 8.0, 6.7, and 12.9 in 3xTg-AD). Under all tested conditions, an important reduction in mitochondrial respiration was observed in synaptic mitochondria from aged WT mice compared with those from young WT mice, and this phenotype was further exacerbated in synaptosomes obtained from the 3xTg-AD animals (WT = 4.4, 3.5 and 7.1 vs. 3xTg-AD = 3.0, 1.6 and 5.1) (Figure 2B, Table 1). Synaptosomal mitochondria from the cerebellum of young animals showed similar responses, but a remarkable reduction in mitochondrial function was observed in synaptic mitochondria from old WT mice, with no further reduction observed in those from old 3xTg-AD mice (Figure 2C, Table 1). In the case of mitochondria from the cerebral cortex and hippocampus, a coupled pattern of oxygen consumption was observed, while synaptic mitochondria from the cerebellum seemed to be uncoupled at old age independent of the transgenic condition. To further study mitochondrial dysfunction, we also measured mitochondrial membrane potential using the Safranin O fluorescent probe. According to the bioenergetics results, we also found a significant reduction in this parameter in aged synaptosomes from the WT cerebral cortex and hippocampus (30% and 40%, respectively) that was more prominent in aged 3xTg-AD animals than in young 3xTg-AD animals (65% and 70%, respectively) (Figure 2D,E), suggesting compromised ATP production in synaptosomes from aged animals [31]. Similar to the effects revealed by the oxygen consumption experiments, the membrane potential of synaptosomal mitochondria isolated from the cerebellum was decreased by aging, and no further reduction was observed in old 3xTg-AD mice (Figure 2F).

### 2.3. Mitochondrial Fission Proteins Are Activated by Aging in 3xTg-AD Mice

**Figure 3:**
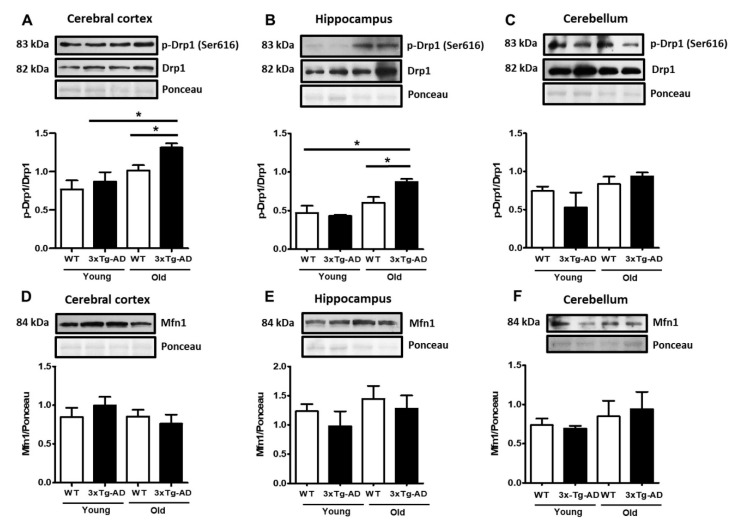
Changes in mitochondrial fission and fusion proteins. Representative Western blot and densitometric analysis of Drp1 and p-Drp1 in cortical (**A**), hippocampal (**B**), and cerebellar synaptosomes (**C**). The contents of Mfn1 in cortical (**D**), hippocampal (**E**), and cerebellar (**F**) samples are shown. Bars represent the densitometric analysis of proteins normalized to Drp1 or Ponceau expression and represent the mean ± SEM of 3–4 independent experiments. Student’s t test * *p* < 0.05.

As mitochondrial membrane depolarization disrupts the mitochondrial fusion/fission balance, we measured the total contents of the active form of the mitochondrial fission factor dynamin-related protein 1 (p-Drp1) and the fusion protein Mfn1. A slight increase in p-Drp1 occurred only in the aged synaptosomes compared with the young synaptosomes from the cerebral cortex and hippocampus, and this effect was statistically significant in the aged 3xTg-AD mice in both regions (80% and 70%, respectively) (Figure 3A–C). To further analyze changes in mitochondrial dynamics, we examined whether mitofusin (Mfn1) from synaptosomes was affected by aging. Western blot experiments revealed high basal levels of this protein, but no significant changes were detected at any age in WT and 3xTg-AD mice (Figure 3D–F).

### 2.4. Aβ and Tau Accumulation in Synaptic Mitochondria

**Figure 4:**
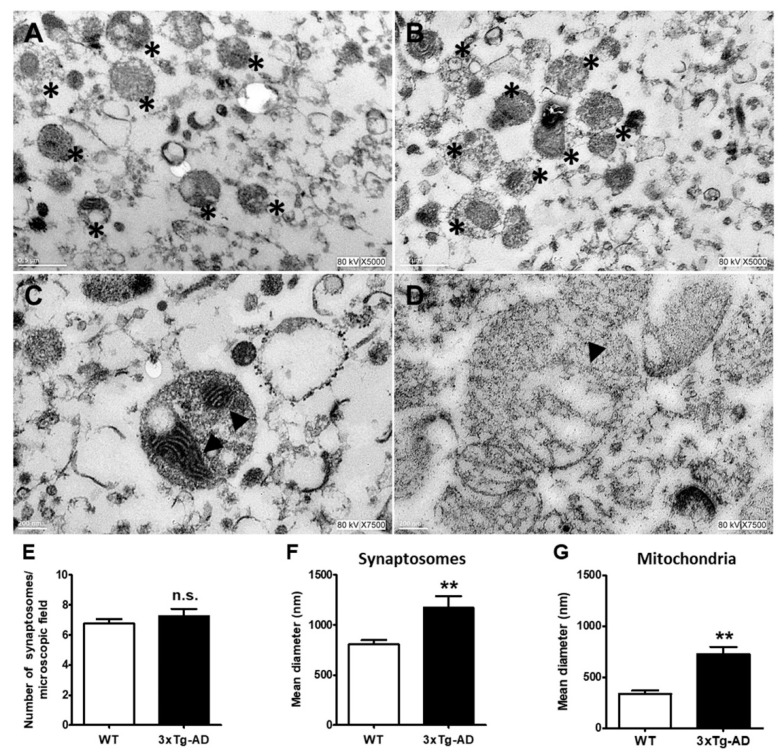
Ultrastructural changes in middle-aged hippocampal synaptosomes. Representative electron microscopy images of synaptosomes (*) from WT (**A**) and 3xT-AD mice (**B**). Changes in synaptic and mitochondria size in WT (**C**) and 3xTg-AD mice (**D**) are depicted. Mitochondria are marked with arrowheads. Bars represent the mean ± SEM of 3–4 synaptosomal random sections from each experimental condition from 3 independent experiments. Synaptosomes number (**E**), synaptosome diameter (**F**), and mitochondrial diameter (**G**). Scale bar = 200 nm. Student’s t test, ** *p* < 0.01; n.s. = no statistically significant difference.

Based on the information that mitochondria may accumulate aggregated proteins, we decided to analyze morphological changes in synaptic mitochondria with age and the accumulation of Aβ and tau in 3xTg-AD mice in the most affected region in early stages of AD. In Figure 4, representative electron microscopy images of synaptosomes obtained from the hippocampus of old WT and 3xTg-AD mice are depicted and quantified (Figure 4E). As shown, synaptosomes from 3xTg-AD mice exhibited larger synaptic diameters and larger mitochondria than those from WT mice (Figure 4A–D). These changes can be clearly observed in the corresponding graphs (Figure 4F,G). 

**Figure 5:**
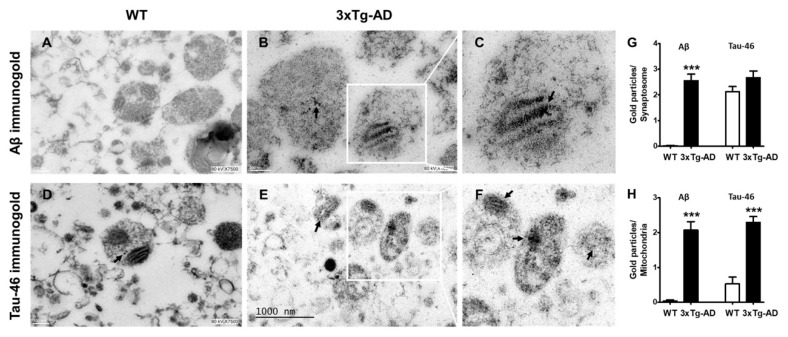
Increased accumulation of Aβ and tau in synaptosomes from middle-aged WT and 3xTg-AD mice. Representative immunogold electron microscopy images of Aβ1-42 and tau labeled with a gold-conjugated antibody (12 nm size) demonstrating Aβ and tau accumulation (black arrows). Hippocampal synaptosomes from WT (**A**,**D**) and old female 3xTg-AD mice (**B**,**E**). (**C**,**F**) are magnifications of **B**,**E**. Quantification of immunogold particles is depicted (**G**,**H**). Student’s t test, *** *p* < 0.001.

To further analyze whether synaptosomes from 3xTg-AD mice accumulate Aβ and tau in old age, we performed immunogold labeling experiments. We found a significant accumulation of immunogold particles bound to Aβ mainly localized inside synaptic mitochondria. No Aβ particles were observed in synaptosomes from WT mice (Figure 5, compare A with B and inset in C). Interestingly, few immunogold particles directed against tau protein were observed in WT animals (arrow), but prominent accumulation was evident in synaptosomes from old 3xTg-AD animals (Figure 5, compare D with E and inset in F).

## 3. Discussion

Altered mitochondrial function is thought to be an important factor associated with synaptic vulnerability during aging. However, it is not entirely understood whether a decline in the number and quality of mitochondria or if the accumulation of toxic molecules inside mitochondria are the main factors responsible for this decay.

Here, we reported that age is the main factor associated with the decline in synaptic mitochondrial function and that the accumulation of toxic compounds, such as tau and Aβ, that occur in the 3xTg-AD model may exacerbate this decline. It was noteworthy that the observed decay in synaptic mitochondrial function in wild-type mice seems to be prominent at the middle age correlating with the impairment of long-term potentiation (LTP) [8] and memory decline [32] without significant loss of presynaptic markers [7]. 

Another important finding was the observation that synaptosomes from the cerebellum are particularly vulnerable to the aging process, because no additional decay in mitochondrial function was observed in the transgenic animals, in accordance with the finding that few neurofibrillary tangles and amyloid plaques are present in this region, even in late AD [33,34]. Thus, we provide evidence supporting the notion that in cerebellar synaptic mitochondria, respiratory capacity and coupling control decline significantly with increasing age independent of the presence of AD-altered proteins. Similarly, several studies have shown that the cerebellum is highly susceptible to aging and is one of cerebral regions in which neuronal loss and significantly reduced synapse density have been reported in aged rats and humans [35,36]. The susceptibility of the cerebellum to age-related pathology has been demonstrated in a group of AD patients with the rare PS1-E280A mutation, which results in specific Purkinje cell loss and mitochondrial damage prior to Aβ accumulation [34], indicating that mitochondrial dysfunction is a central participant in the susceptibility of the cerebellum to aging and AD.

Additionally, we found that under basal conditions, mitochondrial respiratory capacity was similar in synaptosomes purified from young WT and transgenic animals (3 months old). In all cases, the oxygen consumption rate of intrasynaptosomal mitochondria was consistently reduced in the presence of oligomycin, and it was activated by the mitochondrial uncoupler of oxidative phosphorylation, FCCP, in the three brain regions. These results indicate that, in general, intrasynaptic mitochondrial function was normal and that the mitochondria were well coupled. However, in synaptosomes from old WT mice, a significant decline in the mitochondrial respiratory capacity and coupling control decline was observed, suggesting that altered synaptic bioenergetics is mainly associated with brain aging and that a reduction in oxygen consumption was probably associated with a reduced number of healthy mitochondria present in the aged synapse. In this sense, it has been proposed that during aging, a reduction in synaptic mitochondria may be the result of disrupted anterograde axonal transport, a condition that is exacerbated in AD due to microtubule destabilization [37,38]. Thus, mitochondrial functionality at the synapse depends not only on the presence of healthy mitochondria but also on the transport rate of these organelles from the neuronal body.

Abnormal mitochondrial dynamics is an early event linked to neurodegeneration. In particular, Drp1, a key fission protein, has been associated with AD progression [19,39,40,41]. We found that synaptic aging was accompanied by the increased expression of the activated form, p-Drp1, without significant changes in the total content of Drp1. This overexpression was exacerbated in synaptosomes from animals that accumulate Aβ and tau. We do not know which mechanism is involved in Drp1 activation by phosphorylation during aging, although one of the kinases involved in the Ser616 phosphorylation of this protein is the calcium-calmodulin-dependent kinase II (CaMKII) [42,43]. Considering that aging is associated with diverse defects in Ca^2+^ homeostasis (revised in [44]), it is plausible that Ca^2+^ overload at synapsis could account for the activation of CaMKII and consequently the phosphorylation of Drp1. This is an interesting possibility to be analyzed in the future. In addition, several studies have reported that the interaction between Drp1 and Aβ and p-tau enhances GTPase-Drp1 enzymatic activity [22,45]. The consequence of Drp1 activation is mitochondrial fragmentation, which also involves the kinase Pink1. Interestingly, the inhibition of Drp-1 ameliorates mitochondrial fragmentation and improves cognitive performance in the APP/PS1 transgenic model of AD [19]. Our results showed morphological changes consistent to mitochondrial swelling that do not exclude previous mitochondrial fragmentation. The mitochondrial changes observed in synaptosomes from the were similar to that observed in synaptosomes from the rat hippocampus exposed to Aβ and after internal Ca^2+^ mobilization [29].

The progressive dysfunction of mitochondria may also impact the synaptic accumulation of toxic proteins, because mitochondria possess the ability to sequester and degrade damaged proteins [46,47]. Thus, the decline in mitochondrial function in aging and in AD could contribute to diminished cellular proteostasis that exacerbates the accumulation of protein aggregates, as we have observed in mitochondria isolated from 3xTg-AD mice.

Data from the present study demonstrate the prominent role of aging in synaptic mitochondria vulnerability in different brain regions and the role of the associated pathological condition that exacerbates this damage in old individuals and that may further promote synaptic dysfunction. These data suggest that bioenergetic restoration maneuvers could be a useful therapeutic tool to preserve synaptic function during aging and at early the steps in AD.

## 4. Material and Methods

### 4.1. Animal Model

The female 3xTg-AD mice [48] and B6129SF2/J wild-type (http://jaxmice.jax.org/strain/101045.html) mice used in this study were 2–3 months old (3-month-old group; young) or 9–11 months old(10-month-old group; middle-aged) at which point the mice exhibited cognitive decline and expressed significant levels in AD markers, as reported by Belfiore and et al. [49]. Animals were handled with all precautions necessary to avoid suffering in agreement with the ARRIVE guidelines and Regulations for Research in Health Matters (México). The protocol was approved by the local Animal Care Committee (protocol number 179, approved on 11 November 1917). Mice were housed under a 12/12 h light/dark cycle with water and food ad libitum. To verify the homozygosity of the transgenic female mice, genotypification was carried out for each animal using the following primers: APP-tau, 5tauRev (5’-TCCAAAGTTCACCTGATAGT-3’); APP internal (5’-GCTTGCACCAGTTCTGGATGG-3’); Thy12.4 (5’-GAGGTATTCAGTCATGTGCT-3’). To amplify PS1, PS1-K13 (5’-CACACGCACACTCTGACATGCACAGGC-3’) and PS1-K15 (5’-AGGCAGGAAGATCACGTGTTCCAAGTAC-3 ‘) were used.

### 4.2. Synaptosomal Preparation

At the appropriate age, the mice were sacrificed by decapitation, and the frontal cortex, hippocampus, and cerebellum were dissected. Synaptosomes were purified following the procedure described by Löscher et al. [50], with slight modifications [51]. In brief, the brain regions from both hemispheres were dissected on ice, homogenized in a solution containing 0.32 M sucrose, and centrifuged at 4500 rpm for 10 min (4 °C). The supernatant was carefully placed on 1 mL of 1.2 M sucrose and then centrifuged at 50,000 rpm (4 °C) for 20 min. The gradient interphase was carefully collected and diluted with 0.32 M sucrose to a final volume of 2 mL. The diluted suspension was then layered onto 1 mL of 0.8 M sucrose and centrifuged for 20 min at 50,000 rpm. This procedure yielded a synaptosomal pellet that was resuspended in 1 mL of Locke’s solution containing (in mM) 154 NaCl, 5.6 KCl, 2.3 CaCl_2_, 1 MgCl_2_, 3.6 NaHCO_3_, 5 glucose, and 5 HEPES, pH 7.2. Aliquots of 100 μL of synaptosomes were incubated for 90 min at 37 °C in low K^+^ (5 mM) solution. Using this method, synaptosomes isolated from the cerebellum corresponded to the fraction called “small synaptosomes” that come mainly from granule cell synaptic contacts [52,53].

### 4.3. Evaluation of Mitochondrial Redox Activity in Synaptosomes

The method employed in the present study was previously described to evaluate metabolic activity in cultured cells through the MTT colorimetric assay that converts the tetrazolium salt 3-(4,5-dimethylthiazol-2-yl)-2-5-diphenyltetrazolium bromide (MTT) to formazan crystals by oxidoreductase mitochondrial enzymes [54] and was modified from our previously described method [29]. The MTT compound was dissolved in phosphate-buffered saline (PBS) to a concentration of 5 mg/mL, and 10 μL was added to 100 µL of synaptosomes (90 µg of protein) after 30 min and incubated for an additional 90 min. Then, synaptosomes were centrifuged, and the pellet was solubilized with 2-isopropanol acid (100 µL). The resulting colored solution was quantified at 570 nm using a spectrophotometer (Pharmacia Biotech, Gaithersburg, MD, USA). The results are expressed as relative units of MTT reduction. Values are mean ± SEM of 3–4 measurements made in duplicate.

### 4.4. Synaptosomal Mitochondria Bioenergetics

The oxygen consumption rate (OCR) of synaptic mitochondria was measured using a Strathkelvin 782 oximeter (Warner/Strathkelvin Instruments, Holliston, MA, USA) with a Clark-type electrode immersed in a 1 mL chamber with a PolyScience bath (Model 9000, Niles, Il, USA). In brief, synaptosomes were resuspended in 700 μL of respiration buffer (100 mM trehalose, 10 mM KH_2_PO_4_, 10 mM HEPES, 1 mM EGTA, 1.3 mM MgCl_2_, 1.2 mM Na_2_SO_4_, 54 mM NaCl, 1 mg/mL bovine serum albumin (BSA), pH 7.2). Seventy-five microliters (approximately 70 μg of protein) of synaptosomal solution from each brain region was diluted with 75 μL of the respiration buffer. Oxygen consumption was measured in duplicate over 20 min under three different conditions: basal consumption in the presence of 10 mM glucose, 6 μM oligomycin, an ATP synthase inhibitor, or 1 μM carbonyl cyanide-4-(trifluoromethoxy)phenylhydrazone (FCCP), a protonophore. The O_2_ consumption in nmol/min/mg protein was calculated from the slope.

### 4.5. Mitochondrial Membrane Potential

Mitochondrial membrane potential was evaluated according to changes in Safranin O fluorescence at a wavelength of 495/586 nm (em-ex). All experiments were performed in 96-well plates. In each well, 75 µL of respiration buffer (100 mM trehalose, 10 mM KH2PO4, 10 mM HEPES, 1 mM EGTA, 1.3 mM MgCl_2_, 1.2 mM Na_2_SO_4_, 1 mM NaCl/BSA 1 mg/mL) plus 1 µM Safranin O were added and incubated for 10 min at 37 °C. Subsequently, 75 µL of synaptosomal solution (70 μg total protein for cerebral cortex and cerebellum and 65 μg for hippocampus) was added and incubated for 15 min at 37 °C. At the end of this incubation, 1 µM FCCP was added for an additional 40 min. The mitochondrial membrane potential was obtained by subtracting the fluorescence after decreasing the potential of the basal fluorescence. 

### 4.6. Electrophoresis and Immunoblot

The total amount of synaptosomal protein was measured with a BioRad DC^TM^ protein assay kit (Richmond, CA, USA). Twenty micrograms of protein were loaded in a 10% sodium dodecyl sulfate (SDS) polyacrylamide gel and subsequently transferred to polyvinylidene fluoride (PVDF) membranes. After 2 h of incubation in PBS/BSA, the blots were incubated with the fission protein antibody Drp1 (1:1000, Abcam, Cambridge, MA, USA), the activated form pS616-Drp1 (1:500, Cell Signaling, Danvers, MA, USA) or the fusion protein antibody Mfn1 (1:1000, Abcam, Cambridge, MA, USA). The antibodies were left overnight at 4 °C, and then, the blots were washed three times with PBS-Tween 20 0.1% (10 min each). Then, the blots were incubated with horseradish peroxidase-conjugated secondary antibodies using an antirabbit IgG (1:12,000, Santa Cruz Biotechnology, Dallas, TX, USA) or anti-mouse IgG (1:15,000, Santa Cruz Biotechnology, Dallas, TX, USA) for 2 h at room temperature. The membranes were processed using the chemiluminescence ECL substrate (Millipore, Madison, WI, USA) and revealed with a Kodak X-Omat. The values obtained for each band were divided by the values obtained for Drp1 or ponceau staining, resulting in a ratio. Western blots bands were densitometrically analyzed by ImageJ software.

### 4.7. Electron Microscopy Analysis

Five hundred micrograms of synaptosomal protein from the hippocampus was centrifuged and the pellet was fixed in 3% glutaraldehyde, pH 7.4 for 30 min. At the end of this period, the pellet was rinsed in PBS and postfixed in 1% osmium tetroxide. Sections were embedded in epoxy resin and cut with a diamond knife (Ultracut Reichert Jung, Buffalo, NY, USA). For Aβ or tau immunogold labeling, the grid containing synaptosomes was carefully washed with distilled water and blocked with PBS/BSA 5%/horse serum 0.3% for 30 min at room temperature. The grids were incubated with 1 drop of anti-Aβ 1-42 (1:50, Millipore, Madison, WI, USA) or anti-tau (Tau-46 1:50, Cell Signaling, Danvers, MA, USA) overnight at 4 °C. Then, the grids were washed 3 times with PBS and incubated with the secondary anti-mouse IgG-gold (1:30, particle size 12 nm, Abcam, Cambridge, MA, USA) for 1 h at room temperature. At the end of this period, the grids were washed with distilled water, stained with uranyl acetate, and observed under a transmission electron microscope (JEOL 1200 EX-II, Peabody, MA, USA). For each age group, 2–3 different animals were analyzed, and 3 micrographs (8000× and 25,000×) showing 3–4 synaptosomal sections from each individual from each experimental condition were randomly chosen.

### 4.8. Statistical Analysis

The results are expressed as the mean ± SEM. Comparisons between groups were performed using unpaired Student’s t test or ANOVA followed by Tukey’s test, as indicated in each figure legend. *p* < 0.05, *p* < 0.01, or *p* < 0.001 was considered significant. GraphPad Prism 5.01 (San Diego, CA, USA) was used for graphics and statistical analysis.

## Figures and Tables

**Table 1:** Oxygen consumption of synaptosomes from cortex, hippocampus, and cerebellum of female WT and 3xTg-AD mice (nmol O_2_/min/mg protein).

	Young		Old	
	WT	3xTg-AD	WT	3xTg-AD
Cortex				
Glucose	8.002	6.969	3.563 **	2.531 **^&^
Oligomycin	5.393	3.869	2.102 **	1.626 **
FCCP	12.800	14.130	4.093 **	3.318 **
Hippocampus				
Glucose	9.801	8.022	4.458 **	3.036 **^&^
Oligomycin	6.798	6.748	3.515 **	1.594 **^&^
FCCP	13.742	12.907	7.120 **	5.119 **^&^
Cerebellum				
Glucose	7.005	5.843	2.704 ***	2.787 ***
Oligomycin	3.258	4.277	2.601 ***	1.726 ***
FCCP	10.794	14.426	2.993 ***	3.070 ***

Key: ** *p* < 0.01; *** *p* < 0.001 relative to synaptosomes obtained from young WT mice and ^&^ *p* < 0.05 relative to synaptosomes from old WT mice.
